# Spatio-temporal Use of Oral Rabies Vaccines in Fox Rabies Elimination Programmes in Europe

**DOI:** 10.1371/journal.pntd.0003953

**Published:** 2015-08-17

**Authors:** Thomas F. Müller, Ronald Schröder, Patrick Wysocki, Thomas C. Mettenleiter, Conrad M. Freuling

**Affiliations:** 1 Institute of Molecular Virology and Cell Biology, Friedrich-Loeffler-Institut, WHO Collaborating Centre for Rabies Surveillance and Research, Greifswald–Insel Riems, Germany; 2 Institute of Epidemiology, Friedrich-Loeffler-Institut, Greifswald–Insel Riems, Germany; Centers for Disease Control and Prevention, UNITED STATES

## Abstract

In Europe, the elimination of wildlife rabies using oral rabies vaccination [ORV] of foxes for more than 30 years has been a success story. Since a comprehensive review on the scope of the different oral rabies vaccine baits distributed across Europe has not been available yet, we evaluated the use of different vaccine baits over the entire period of ORV [1978–2014]. Our findings provide valuable insights into the complexity of ORV programs in terms of vaccine related issues. More than 10 oral vaccines against rabies were used over the past four decades. Depending on many factors, the extent to which oral rabies virus vaccines were used varied considerably resulting in huge differences in the number of vaccine doses disseminated in ORV campaigns as well as in large spatial and temporal overlaps. Although vaccine virus strains derived from the SAD rabies virus isolate were the most widely used, the success of ORV campaigns in Europe cannot be assigned to a single oral rabies virus vaccine alone. Rather, the successful elimination of fox rabies is the result of an interaction of different key components of ORV campaigns, i.e. vaccine strain, vaccine bait and strategy of distribution.

## Author Summary

Oral rabies vaccination [ORV] is the pre-eminent example of successful vaccination of wildlife populations which has resulted in virtual elimination of fox-mediated rabies from large parts of Western and Central Europe. This achievement is unprecedented in history. Attractive species-specific baits, efficient vaccination strategy, and highly potent and safe oral rabies vaccines have been instrumental for the success. Numerous oral rabies vaccines for wildlife have been developed over the past four decades. However, information on the use of those vaccines in operational ORV programs in Europe that exists has until now not been fully publicly accessible. Here, we provide the first comprehensive review of the spatio-temporal use of oral rabies vaccines over the entire period of ORV [1978–2014]. Although ‘first generation’ vaccine virus strains derived from the SAD rabies isolate are the most widely used, the results do not support significant differences in efficacy of these strains under field conditions nor can success of ORV be attributed solely to one vaccine type. In contrast, vaccination strategy is of major importance. Our analyses could help in the development of adequate rabies control strategies by documenting the importance of vaccination strategy, in which oral rabies vaccines are only one contributing component among other parameters.

## Introduction

Elimination of infectious diseases from wildlife populations is a major challenge. Thus, until a few decades ago, elimination of wildlife-mediated rabies from Europe was considered wishful thinking. This paralleled the results of early conventional control measures aimed at stopping the expansion of fox rabies in Europe [1, 2]. Almost all attempts to reduce the fox population below a threshold, where the chain of infection would be interrupted and the epidemic would fade out, using intensive culling, poisoning, and trapping failed [3]. Moreover, scientists, veterinary and public health authorities came to understand that focusing on reduction of the fox population evidently was counterproductive as it disrupted the social and spatial organization of foxes, thereby increasing contact rates and disease incidence [3, 4].

The development of attenuated oral rabies virus vaccines in the second half of the 20th century provided a unique opportunity to target elimination of the infection circulating in European wildlife [5, 6]. Concerted inter-sectorial applied research activities in a few European countries such as Switzerland, Germany and France pioneered the basic techniques for a suitable strategy for oral rabies vaccination (ORV) of foxes. These included the type of baits, timing of vaccination campaigns, bait delivery densities, adequate modes of bait distribution, and duration and monitoring of ORV campaigns [2, 7]. What started as a small but ground-breaking field trial in Switzerland in 1978 [8] soon became the most widely-used model for controlling and eliminating a zoonosis in its wildlife reservoir host. Triggered by promising results of first field trials in the 1980s and accelerated by a strong political commitment of European governments and the co-financing policy of the European Union (EU) for member states and neighboring non-EU countries [9], many rabies affected European countries implemented long-term ORV programs [10]. However, it was not until further scientific and technical achievements, e.g. the development of manufacturing methods for baits and the application of computer supported automatic aerial distribution equipment for baits were made that large-scale vaccination campaigns became possible [2, 11].

Despite perceived challenges and individual setbacks at national or international levels [10, 12], the current rabies situation provides an impressive testimony of the efficiency and future potential of wildlife vaccination [2, 13]. Within the past 30 years the overall rabies incidence in Europe decreased by approximately 80% compared to the peak year 1984 during which 24,315 rabies cases were reported (www.who-rabies-bulletin.org). Furthermore, the disease was completely eliminated from Western and Central Europe [2, 13, 14]. The proportion of landscape area ever affected by rabies and an index capturing the size and overlap of successive ORV campaigns were identified as factors having statistically significant effects on the number of campaigns required to control and eliminate rabies [13].

Highly potent and safe oral rabies vaccines have been the key for the elimination of wildlife rabies in Europe and several regions of North America [10, 15]. All rabies vaccines used for oral immunization of wildlife in Europe are based on live replication-competent vaccine viruses [15]. The first generation vaccines were attenuated rabies viruses developed by conventional in vivo and/or in vitro serial passaging of virulent field virus isolates resulting in e.g. SAD Bern, SAD B19, SAD P5/88, Vnukovo-32, or RV-97 [16–19]. The second generation was developed by selection of monoclonal antibody escape mutants, e.g. SAD VA1, SAG1, or SAG2 [20–23]. Later, site-directed mutagenesis [reverse genetics] led to the development of a third generation of oral rabies vaccines, e.g. ERA G333 [24]. Finally, a recombinant vaccinia virus expressing the rabies virus glycoprotein from the ERA strain [V-RG] [25, 26] has been used. The progenitor of almost all attenuated rabies virus-based vaccines currently in use in Europe is the vaccine virus strain SAD Bern [27], a derivative of the original SAD (Street Alabama Dufferin) virus isolated from a rabid dog in 1935 in the US [28].

The limited available information on the use of those vaccines in ORV programs in Europe [7, 29, 30] resulted in speculations whether the efficiency of ORV programs in Europe can be attributed to a single vaccine only or whether certain vaccines are more efficient than others under field conditions. However, a comprehensive review of the scope of oral rabies virus vaccines distributed across Europe had not been available. We therefore evaluated the spatio-temporal use of different oral rabies virus vaccines in individual European ORV programs over the past 37 years.

## Materials and Methods

### Study region and ORV approach

**Fig 1:**
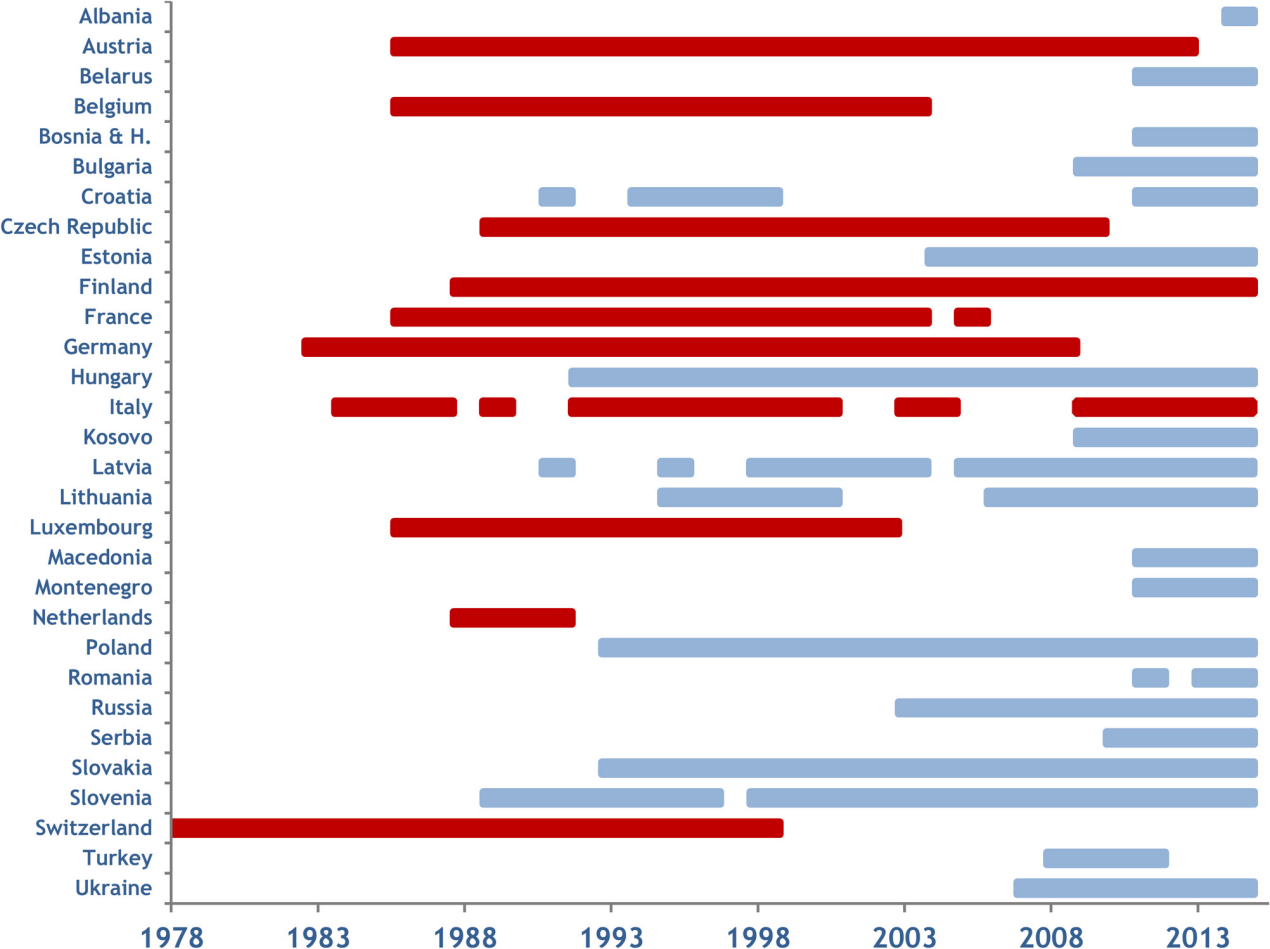
Implementation of ORV programmes in Europe (1978–2014). The year of (re-) implementation, duration and the year of cessation of ORV programmes including early field trials in several European countries is depicted. Red coloured bars show countries which achieved a rabies free status due to ORV and blue coloured bars represent countries in which ORV programmes are still implemented.

The study region encompassed all countries in Europe that implemented ORV programs on their territories between 1978 and 2014 (Fig 1). The standard ORV approach applied typically comprised of (i) performance of ORV campaigns twice a year (spring and autumn), (ii) an average bait density of 20–25 baits/km^2^, (iii) aerial and manual (mainly at the beginning of ORV, later complementary) distribution of vaccine baits, and (iv) a flight line distance of 500 to 2000 m in case of aerial distribution of baits [13]. In a few exceptions ORV campaigns were only conducted once a year (Italy, 1984) or additional campaigns were conducted either in summer or in winter (France, Germany 2005, Italy 2009) or at short intervals (Germany, 2005; Italy, 2010) [31,32].

### Data collection

As part of the terms of reference as a WHO Collaborating Centre for Rabies Surveillance and Research, data related to ORV programs of individual European countries for the past 37 years were collected from three different sources including (i) information provided by veterinary or other competent authorities (list of contributors to the WHO Rabies Bulletin Europe, http://www.who-rabies-bulletin.org) upon request, (ii) presentations of members states given at meetings the Standing Committee on the Food Chain and Animal Health—Section: "Animal health and animal welfare" (SCFCAH) [http://ec.europa.eu/food/committees/regulatory/scfcah/animal_health/index_en.htm], and (iii) official websites of competent authorities of European countries concerned. For each ORV campaign (spring, summer, autumn, winter or other) carried out during the observation period data comprised information on the size of vaccination areas, the timing of vaccination campaigns, bait density, mode of bait distribution, and oral rabies vaccine strains used. The size and location of individual vaccination areas was either requested as shape files or, if not available, as scanned maps from publications or presentations [13].

### Data analysis

For countries that implemented ORV programs during the review period we established a GIS database with individual campaign based datasets using ArcGIS software [Esri Inc., version 10.2., Redlands, California, USA]. Maps of vaccination areas that were not available as shape files were digitalized, converted into the GIS databases as previously described [33], and subsequently sent to competent authorities or rabies experts of the respective European countries for validation and revision, if necessary. Information on the oral rabies vaccines used was assigned to any individual vaccination area or sub-region (national units of territories) therein. Data of the entire observation period were subsequently stratified, compiled and displayed in maps. Using ArcGIS analysis tools we calculated the size of the area exclusively vaccinated in Europe with a single oral rabies vaccine or with multiple vaccines by considering vaccine combinations. For each oral rabies vaccine used during the observation period we also calculated the area in which it was used. The total number of vaccine baits for each individual vaccine distributed in Europe was computed based on the size of the cumulative vaccine-specific vaccination areas and an assumed average bait density of 20 baits/km^2^.

## Results

During the past 37 years, 30 European countries implemented ORV programs. In autumn 1978, Switzerland conducted the first European ORV campaign ever; the last country to join efforts to eliminate fox-mediated rabies was Albania in 2014. In seven countries, ORV programs were discontinued and re-initiated either due to re-infection, establishment of a vaccination belt (*cordon sanitaire)* as a result of unfavorable rabies situation in neighboring regions, or budget constraints. During the review period 10 countries successfully eliminated fox and raccoon dog-mediated rabies from their territory using ORV (Fig 1).

**Fig 2:**
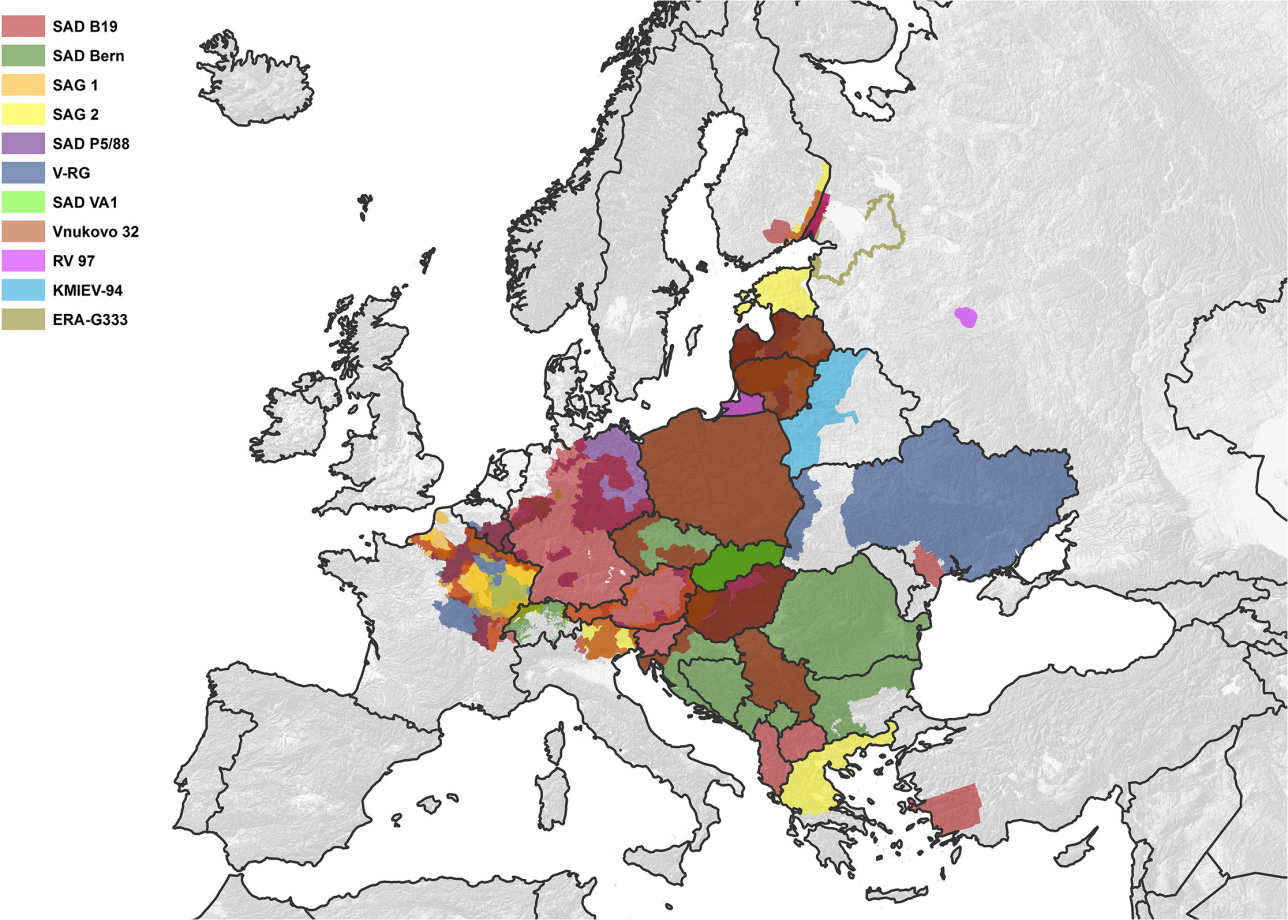
ORV effort and oral vaccine strains against rabies. Spatial extent of ORV area showing the spatial distribution of different oral vaccine strains against rabies used between 1978 and 2014. Deviating colours from those assigned to certain vaccines indicate overlapping regions.

Since 1978, the total area ever vaccinated encompassed 2.5 million km^2^, within which a total of ten different attenuated rabies virus-based vaccine strains and one recombinant vaccine were used [Fig 2]. In the early days of ORV in Europe (1978–1984) baits consisted of chicken heads each containing a sachet made of plastic and aluminum foil with 1.8 ml SAD vaccine. Since the development of novel manufactured baits in 1983, later almost all vaccine baits in Europe (SAD P5/88, SAD B19, SAD Berne, V-RG, SAG-1, SAG-2, SAD VA1) have used fish meal as attractant, while the carrier substance differed between the baits (paraffin, polymer, coconut fat). Bait casings usually contained 150 mg of tetracycline as a biomarker.

About 1.52 million km^2^ (60.8%) of the total area comprising different regions in 25 countries were exclusively vaccinated with a single representative of the available 11 vaccines strains. In contrast, in 702,482 km^2^ (28.1%) and 278,415 km^2^ (11.1%) of the area, two and three, or more than three vaccines were distributed, respectively, over the complete time span of ORV in Europe (Tables 1–3).

**Table 1:** Size of ORV areas in European countries in km^2^ exclusively vaccinated with one particular oral vaccine strain against rabies over the entire time span of ORV.

	oral vaccine strain against rabies									
country	SAD B19	SAD Bern	SAD P5/88	SAG2	V-RG	SAG1	ERA G333	KMIEV-94	RV 97	total
Albania	27,599	-	-	-	-	-	-	-	-	27,599
Austria	40,484	-	-	-	-	-	-	-	-	40,484
Belarus	-	-	-	-	-	-	-	79,587	-	79,587
Belgium	-	-	-	-	1459	-	-	-	-	1459
Bosnia-Herzegovina	-	48,375	-	-	-	-	-	-	-	48,375
Bulgaria	-	73,096	-	-	-	-	-	-	-	73,096
Croatia	-	38,143	-	-	-	-	-	-	-	38,143
Czech Republic	-	33,988	-	-	-	-	-	-	-	33,988
Estonia	-	-	-	41,783	-	-	-	-	-	41,783
Finland	8956	-	-	5765	-	-	-	-	-	14,721
France	2723	-	-	-	26,913	11,533	-	-	-	41,169
Germany	154,420	-	42,452	-	-	-	-	-	-	196,872
Greece	-	-	-	68,389	-	-	-	-	-	68,389
Italy	1880	-	-	8692	-	-	-	-	-	10,572
Kosovo	-	10,470	-	-	-	-	-	-	-	10,470
Liechtenstein	-	-	-	30	-	-	-	-	-	30
Macedonia	24,260	-	-	-	-	-	-	-	-	24,260
Montenegro	-	13,221	-	-	-	-	-	-	-	13,221
Romania	-	224,006	-	-	-	-	-	-	-	224,006
Russia	-	-	-	-	-	-	19,753	-	5893	25,646
Slovenia	19,014	-	-	-	-	-	-	-	-	19,014
Switzerland	-	11,011	-	231	-	-	-	-	-	11,242
The Netherlands	481	-	-	-	-	-	-	-	-	481
Turkey	37,036	-	-	-	-	-	-	-	-	37,036
Ukraine	13,129	-	-	-	427,213	-	-	-	-	440,342
**Total**	**329,982**	**452,310**	**42,452**	**124890**	**455,585**	**11,533**	**19,753**	**79,587**	**5893**	**1,521,985**

**Table 2:** Size of ORV areas in European countries in km^2^ vaccinated with two different oral vaccine strains against rabies over the entire time span of ORV.

	combinations of oral vaccine strains against rabies													
country	SAD Bern & SAD B19	SAD B19 & SAD P5/88	SAD B19 & V-RG	SAG1 & SAD B19	SAG2 & SAD B19	SAG1 & SAG2	SAG2 & V-RG	ERA G333 & RV-97	SAG1 & V-RG	SAG1 & SAD Bern	SAD B19 & RV-97	SAD B19 & SAD-VA1	SAD Bern & SAG2	total
Austria	-	5106	-	18,302	-	-	-	-	-	-	-	-	-	23,408
Belgium	-	-	10,843	-	-	-	-	-	-	-	-	-	-	10,843
Croatia	12,104	-	-	-	-	-	-	-	-	-	-	-	-	12,104
Czech Republic	39,569	-	-	-	-	-	-	-	-	-	-	-	-	39,569
Finland	-	-	-	-	6862	-	-	-	-	-	-	-	-	6862
France	-	-	21,193	13,842	-	18,147	16,331	-	5451	-	-	-	-	74,964
Germany	-	85,721	-	-	-	-	-	-	-	-	-	982	-	86,703
Hungary	-	8513	-	-	-	-	-	-	-	-	-	-	-	8513
Italy	-	-	-	-	22,091	-	-	-	-	-	-	-	-	22,091
Latvia	20,399	-	-	-	-	-	-	-	-	-	-	-	-	20,399
Liechtenstein	-	-	-	-	-	-	-	-	-	-	-	-	6	6
Lithuania	15,053	-	-	-	-	-	-	-	-	-	-	-	-	15,053
Luxembourg	-	-	2419	-	-	-	-	-	-	-	-	-	-	2419
Poland	291,085	-	-	-	-	-	-	-	-	-	-	-	-	291,085
Russia	-	-	-	-	-	-	-	12,659	-	-	1124	-	-	13,783
Serbia	72,256	-	-	-	-	-	-	-	-	-	-	-	-	72,256
Switzerland	-	-	-	-	-	84	-	-	-	1401	-	-	745	2230
**total**	**450,466**	**99,34**	**34,455**	**32,144**	**28,953**	**18,231**	**16,331**	**12,659**	**5451**	**1401**	**1124**	**982**	**751**	**702,288**

**Table 3:** Size of ORV areas in European countries in km^2^ vaccinated with three or more different oral vaccine strains against rabies over the entire time span of ORV.

country	combinations of oral vaccine strains against rabies	size
France	SAD B19; SAG1; SAG2; V-RG	31,307
Germany	SAD B19; SAD P5/88; SAD-VA1	9529
Switzerland	SAD Bern; SAG1, SAG2	4760
Austria	SADB19, SAG1; SAD P5/88	14,537
Slovakia	SAD-VA1; Vnukovo 32; SAD Bern	45,826
Hungaria	SAD B19; SAD Bern; SAD P5/88; SAG1	78,574
Serbia	SAD B19; SAD Bern; SAG2	769
Lithuania	SAD B19; SAD Bern; SAG1; SAD P5/88	45,791
Latvia	SAD B19; SAD Bern; SAD P5/88; Vnukovo 32	40,527
Russia	ERA G333; RV-97; SAD B19	6371
Liechtenstein	SAD Bern; SAG1; SAG2	110
**Total**		**278,101**

**Fig 3:**
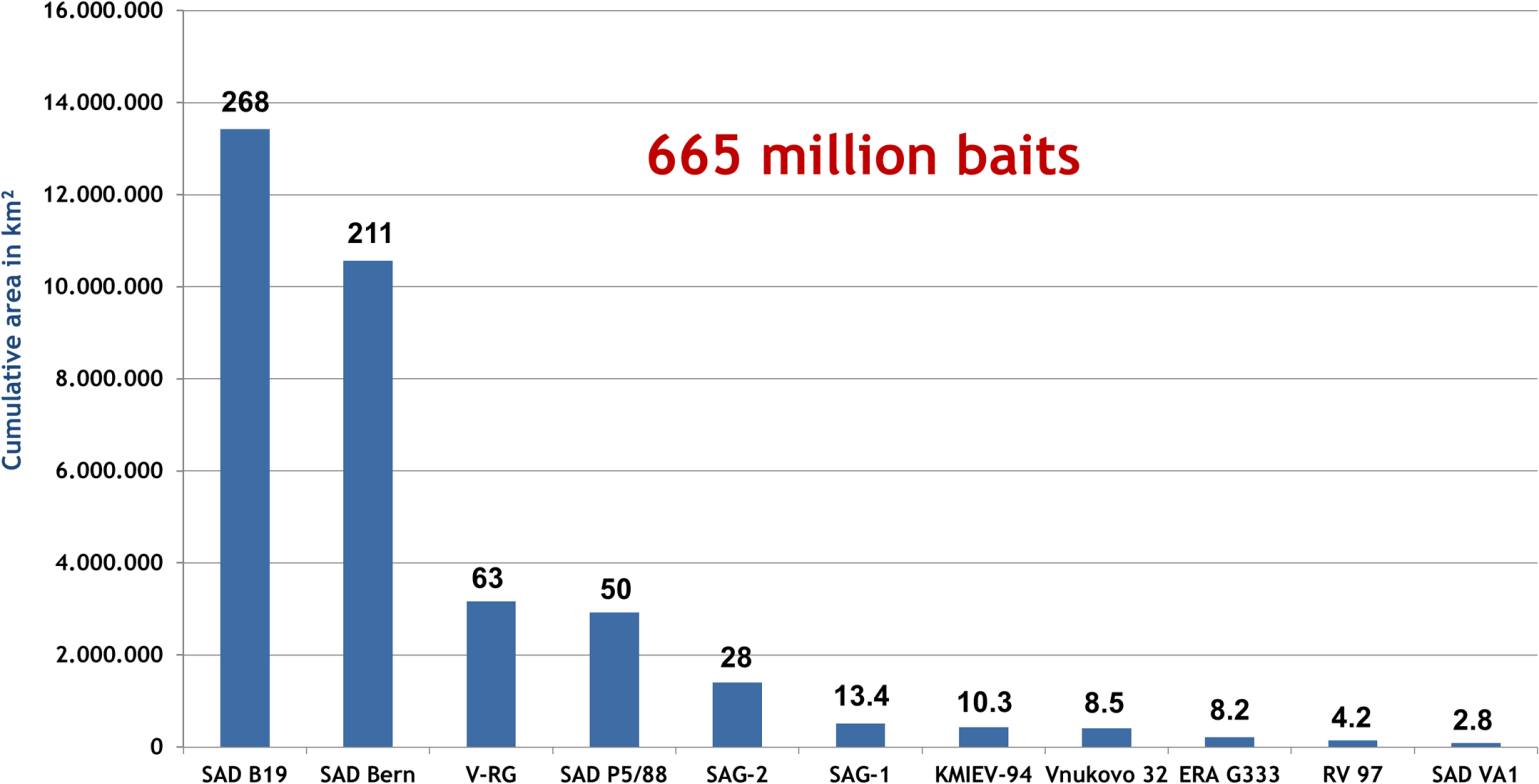
Number of individual vaccine doses disseminated in ORV programmes in Europe between 1978 and 2014. Approximate calculation of the numbers of vaccine doses of different oral vaccine strains against rabies over the past four decades (x axis) based on the cumulative area ever vaccinated with a single vaccine bait (y axis) and an assumed average bait density of 20 baits/km^2^.

**Fig 4:**
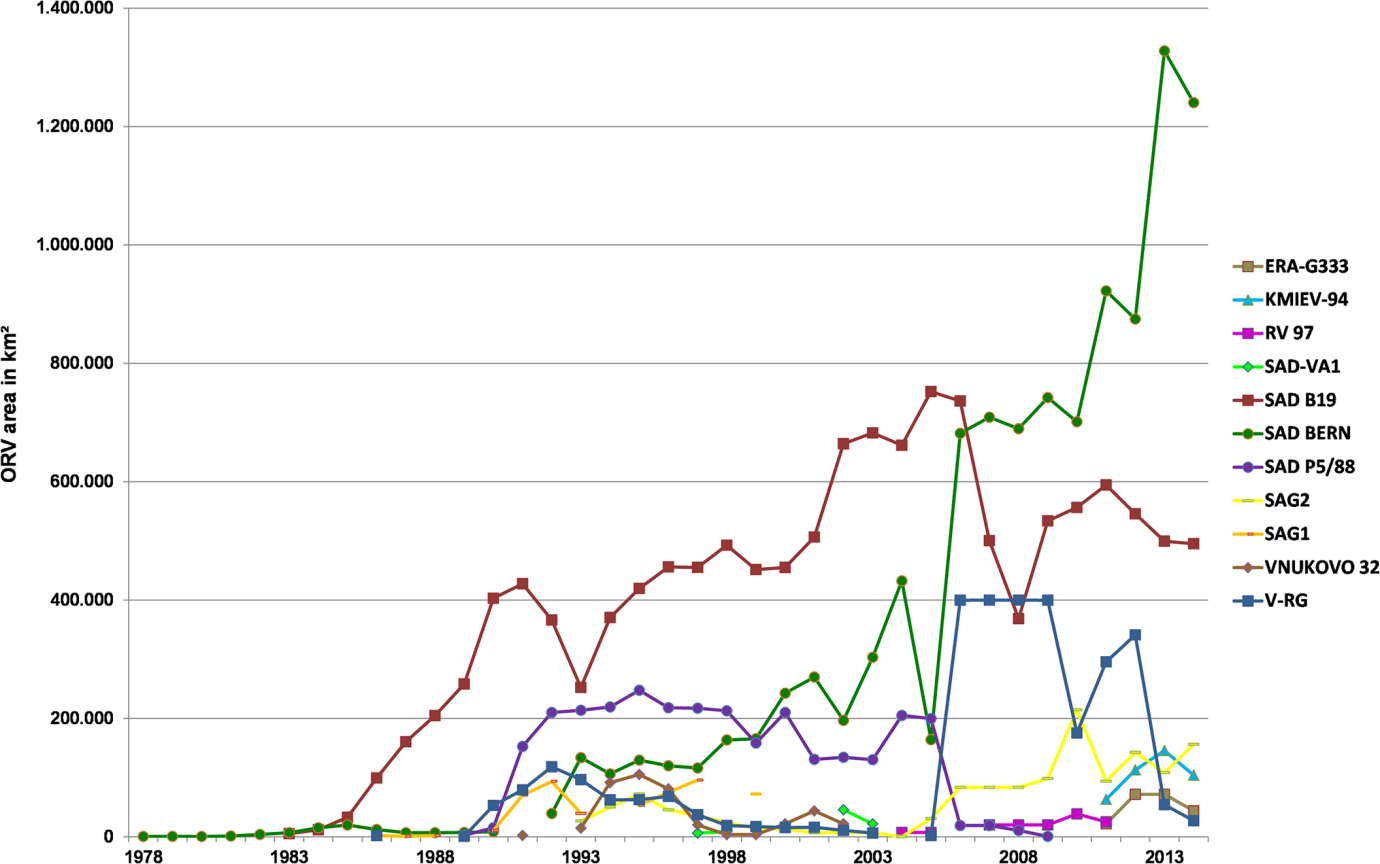
Temporal use of the different oral vaccine strains against rabies over the past four decades.

**Fig 5:**
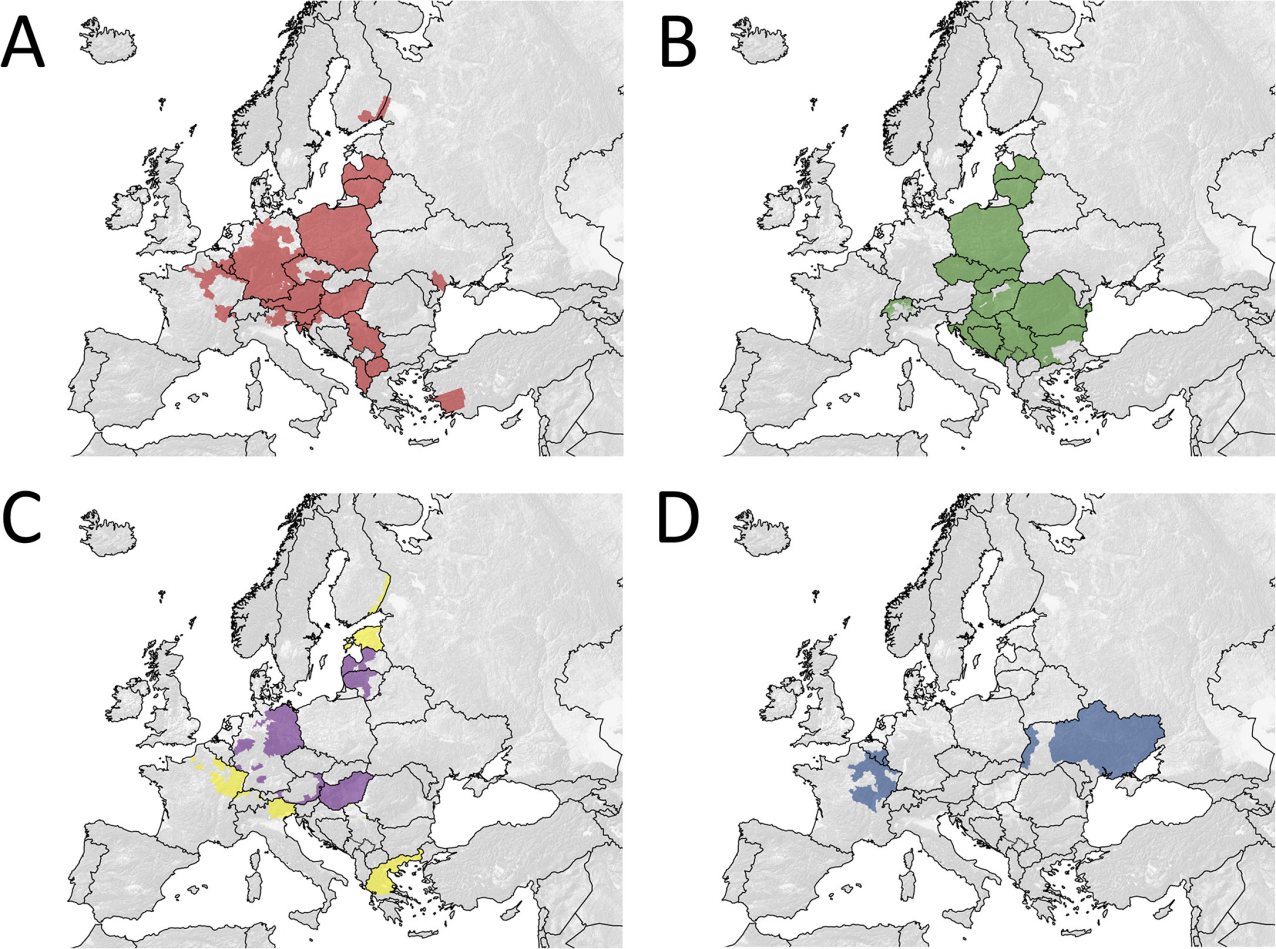
Spatial extent of ORV areas in Europe ever covered with a single vaccine bait of particular oral vaccine strains against rabies during 1978 and 2014. (A) SAD B19, (B) SAD Bern, (C) SAD P5/88 (purple) and SAG2 (yellow), and (D) V-RG.

**Fig 6:**
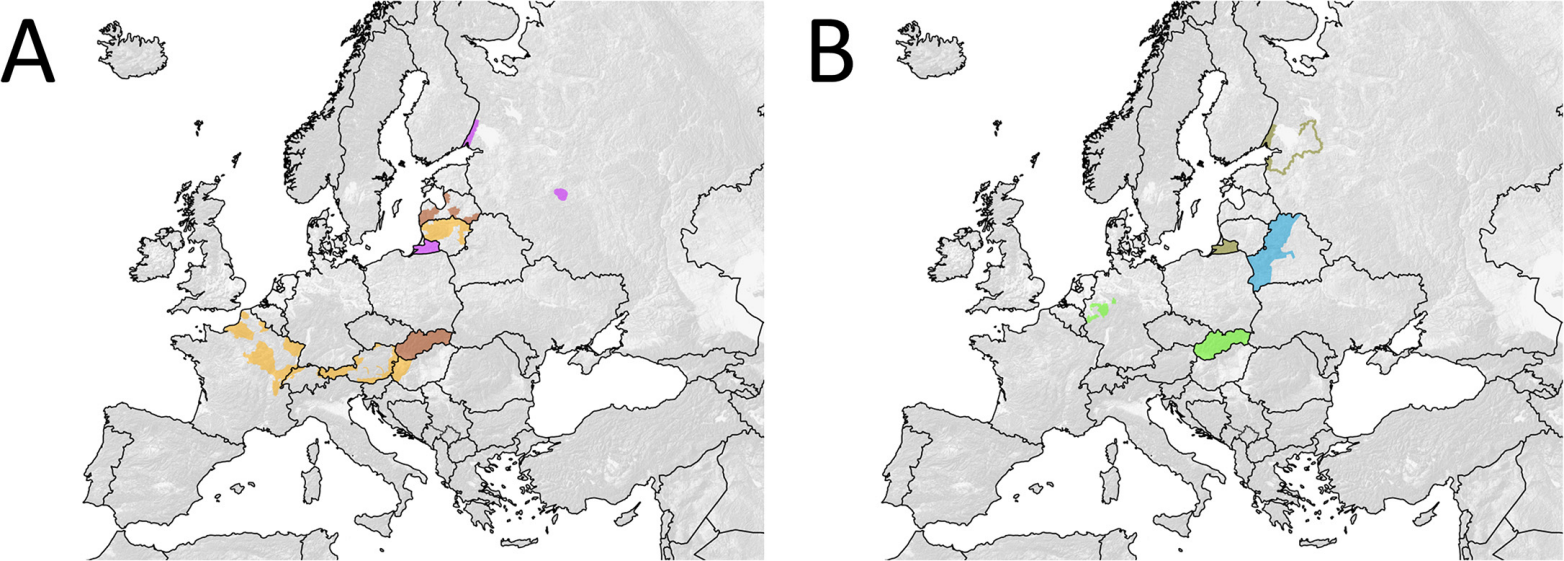
Spatial extent of ORV areas in Europe ever covered with a single vaccine bait of particular oral vaccine strains against rabies during 1978 and 2014. (A) SAG1 (orange), Vnukovo 32 (brown) and RV-97 (magenta), and (B) SAD VA1 (green), KMIEV-94 (blue) and ERA G333 (yellow).

When the size of vaccination areas covered in all individual vaccination campaigns in respective countries between 1978 and 2014 (Fig 1) was summarized, the cumulative vaccination area encompassed 33.25 million km^2^. Assuming an average bait density of 20 baits/km^2^, the estimated total number of vaccine baits distributed between 1978 and 2014 amounts to 665 million (Fig 3). The spatio-temporal use of the different vaccine strains varied considerably. The temporal use of the different vaccine strains is depicted in Fig 4. With a time span of 35 and 32 years of application and an area of 1.96 and 1.12 million km^2^ covered with at least a single campaign by SAD Bern and SAD B19, respectively, these two vaccines were the most widely used throughout Europe. V-RG, SAD P5/88 and SAG2 had been used to a much more limited extent. Although having been authorized for more than 20 years, the area ever vaccinated with vaccine baits of those three vaccine strains comprised 0.54, 0.31 and 0.21 million km^2^, respectively (Fig 5A–5D). In contrast, the remaining vaccine strains were only used at a minor scale during a shorter period of time (<11 years) (Figs 3 and 6A and 6B) with a limited number of baits distributed in the field.

## Discussion

Next to mass dog vaccination which almost exclusively relies on parenteral delivery, the development of ORV allowed the elimination of the infection from wildlife reservoirs [13]. The elimination of fox rabies, e.g. “absence of transmission within a specified area” [34], from large parts of Europe and North America using oral vaccination has been a milestone in the global fight against rabies raising hope for controlling rabies also in other wildlife reservoirs in the near future [10, 35]. Compared to 2010, the area ever vaccinated increased by six hundred thousand km^2^ in 2014, with ten previously endemic or re-infected countries now being rabies-free [13] (Fig 1). Other countries are close to eliminating rabies and will achieve a rabies-free status in the near future. Just recently, Latvia applied for recognition as a rabies free country at the World Organization for Animal Health (OIE) [Oļševskis, personal communication].

Previous descriptive studies on oral rabies virus vaccines in Europe had mainly focused on two vaccine strains and limited time periods, i.e. SAD B19 (1983–1998) and SAG2 (1994–2014) [29, 30]. There had been only one comprehensive attempt to list the number of vaccine baits used for SAD B19, SA1/SAG2, SAD Bern, Vnukovo 32 and V-RG in ORV campaigns in 16 European countries for the years 1978 to 1999. For many countries, however, information for the entire observation period could not be obtained [7]. Here, we provide the first exhaustive analysis to document and analyze the spatio-temporal use of different vaccines during the period of ORV in Europe (1978–2014). The quality of data obtained in this study depended on the willingness of responsible authorities to submit complete sets of data and related information to the European database of the Rabies Bulletin Europe. However, no other substantial and comprehensible database on rabies in Europe is available. Although submission of data and other rabies related information has been entirely relying on voluntary contribution since the beginning, the database is well established and enjoys a very high level of reporting compliance [36, 37]. Like in a previous study, it was difficult to obtain historical information on early field trials with locally developed vaccine strains and baits for Eastern European countries including Belarus, Latvia, Lithuania, Russia, and Ukraine [7]. The use of strain EVMTI VVMKI-71 in early field trials in Latvia [7] could not be confirmed by our sources. However, while we were able to close some of the gaps, the still missing information is unlikely to have any significant impact on the overall outcome of this study.

The number of vaccines used for ORV throughout Europe is higher than previously assumed. While between 1978–2010 a eight different vaccines were reported to have been distributed in countries in which more than four ORV campaigns were conducted during the observation period [13], our study revealed that at least eleven different vaccine virus strains were used for ORV throughout Europe. The new oral rabies virus vaccines strains identified in this study are attenuated rabies virus vaccines RV-97, ERA G333 and KMIEV-94 which have mainly been used in Russia and Belarus, respectively (Figs 2, 3 and 6A and 6B). RV-97 is a derivate of an attenuated “sheep” rabies virus isolate phylogenetically more related to the Japanese group of vaccine strains such as the Nishigara strain [19, 38]. Our data (Fig 6A and 6B) do not match with previous information according to which this vaccine was supposedly used in Kazakhstan, Belarus and Ukraine as well. Unfortunately, no additional information is available [19]. The vaccine strain KMIEV-94 from Belarus was derived from rabies virus strain 71-BelNIIEV VGNKI through serial passages in different cell cultures [39]. No information is available on the origin and genetics of this parental strain, though in contrast, ERA G333 represents the first specifically modified live virus vaccine used in Europe based on site-directed mutagenesis with a good safety profile but reduced efficacy in raccoon dogs as observed in experimental studies [24].

While during numerous ORV field trials it soon became clear that alternative to the labour-intensive and costly preparation of chicken heads was essential, the development of manufactured baits was a decisive breakthrough for large-scale implementation of ORV campaigns in Europe [7]. The so-called Tübingen fox bait was the first manufactured bait used in Europe [40]. Initially, meal from slaughtered livestock [meat, bones, etc.] was used but after the BSE-scarce this component was replaced by fish meal. Although in a previous descriptive study types of baits used in ORV in Europe for the time period 1978 to1991 are listed [7], there is no information on the specific ingredients of the different bait casings as this is part of the intellectual properties of vaccine manufacturers. However, generally, it can be assumed that baits of present available commercial produced vaccines used within EU contain fish meal as attractant with different carrier substances. While data sets on the number and time points of ORV campaigns conducted as well as the size and location of vaccination areas for the entire time span of ORV in Europe (1978–2014) are complete, there remain a few gaps related to bait density applied in different countries during different time periods. This prevented calculation of the exact number of vaccine doses distributed in Europe over the past four decades (Fig 3). Moreover, obtaining such information on country level directly from the manufacturers is one possibility but would only be possible for licensed vaccines. These figures are often considered classified information not to be released. Moreover, the number of vaccine doses delivered to the customer does not necessarily match the number distributed in a given campaign. In fact, not all available vaccine baits are distributed. Frequently, a certain number is kept as emergency stock to then be used in the following campaign Therefore, only the countries themselves know how many baits had actually been distributed. However, the size of the vaccination area directly determines the number of vaccine baits [41, 42]. Considering the fact that depending on experience and epidemiological situation bait densities applied in Europe over the past four decades ranged between 15 and 30 baits/km^2^ with a mean density of 18–20 baits/km^2^ [29, 43, 44] we assumed an average bait density of 20 baits/km for the entire observation period. Hence, the total numbers of vaccine baits presented in Fig 3 are approximate calculations but with a reasonable level of confidence. Considering minimum and maximum bait densities applied in Europe, figures on the number of doses distributed for individual vaccines may slightly vary. However, the relationships between the different vaccine strains will not be affected (Fig 3).

The spatio-temporal use of different oral rabies vaccine strains in Europe over the past four decades is complex (Figs 2–6). While availability of oral rabies vaccines was of prime importance in the initial phase of ORV, subsequently, with more vaccines to choose from, efficacy and safety issues and the development of adequate strategies were prime considerations [7, 10]. With the implementation of a consistent ORV strategy the focus was directed to improvement of vaccine quality, e.g. vaccine titer, stability, and maintenance of cold chain [45]. However, the decisions taken by competent authorities on the use of the different vaccines for ORV programs remained complex and influenced by other factors as well. Vaccine strains such as SAD Bern and SAD B19 were licensed first and quickly established, while the remaining vaccines were developed or licensed relatively late or appeared on the market only recently (Fig 4). Also, based upon strategic decisions of the manufacturers several vaccines were taken off the market and licenses expired (Fig 3). In the European Union for example, vaccines without an EMA (European Medicine Agency) license or marketing authorization in one of the member states (national license) that did not meet requirements of the European Pharmacopoeia, were excluded from ORV programs. Also, there was a tendency of countries to prefer vaccines manufactured in their own country (Germany, France, Czech Republic, Russia and Ukraine).

The use of recombinant vaccinia- virus expressing the rabies glycoprotein is often believed to have substantially contributed to rabies elimination in many European countries [46, 47]. However, in contrast to North America, where this vaccine has been used extensively to control rabies in raccoons, coyotes and skunks [48], V-RG has only been applied to a rather limited scale in Europe [Figs 3 and 5D]. One reason is a general negative public attitude in many European countries on the use of genetically modified organisms, in particular genetically engineered replication-competent agents. Other concerns regarding safety issues and genetic stability of the vaccinia vector virus, its potential to replicate in an extremely wide range of animal hosts, transmission of the recombinant vaccinia virus to humans, and potential recombination with circulating orthopox viruses in reservoir hosts [49, 50], led to a refusal of the majority of European countries on its use. As a result, V-RG was only used in limited parts of four countries including France, Belgium, Luxembourg and Ukraine (Fig 5D).

The most decisive criteria for authorities with regard to vaccines used in ORV programs was the cost of the respective vaccine product. Although ORV programs in EU member states are co-financed up to 75% by the European Union [2, 9, 32], the overwhelming majority of total costs for ORV programs are due to purchase of vaccine baits [13, 41, 51]. Next to differing development and manufacturing costs for individual oral rabies vaccines, pricing is also influenced by tender specifications for implemented ORV programs. Countries usually considered the cost of vaccines as the most relevant criterium for purchase and were used to stimulate vaccine producers to offer reduced pricing. Other countries may have paid more attention to safety issues or technical support during bait distribution. Hence, there is evidence that the price of individual vaccines varied considerably between countries.

The significant success of ORV in controlling and eliminating wildlife rabies in Europe is mainly due to attenuated oral rabies virus vaccines also referred to as “first generation vaccines”, of which the vaccine strains SAD B19 and SAD Bern are by far the most widely used (Figs 3 and 5A and 5B). This descriptive statistic disagrees with international recommendations for ORV of wildlife and dogs suggesting to give preference to vaccines with reduced (non-rabies related) pathogenicity, such as recombinant vaccines (V-RG) or a highly attenuated live virus strain (SAG2), over more pathogenic attenuated live viruses [52]. While attenuated rabies virus vaccine strains, based on experimental data, differ in residual pathogenicity [53, 54], interestingly such a comparison is not possible using field data. Eleven vaccine-associated rabies cases have been described under field conditions in immune-suppressed foxes and also non-target species for SAD Bern, SAD B19, and SAD P5/88 [55–57] resulting in an incidence rate of 1 in 48 million vaccine doses distributed. For each of the remaining seven attenuated oral rabies virus vaccines including the “second generation vaccines” SAG2, however, an equivalent number of vaccine baits has not been disseminated over the past 37 years (Figs 3 and 5C). The use of baits to eliminate rabies from endemic areas as compared to establishment of a vaccination belt (*cordon sanitaire*) to prevent re-infection of a rabies-free area also influences the likelihood of these adverse incidents as the chance of detecting vaccine associated cases is higher in the latter case. In any case, the reported SAD derived rabies cases apparently did not have any epidemiological relevance as the vaccine viruses did not become established in wildlife [56].

Efficacy of oral rabies virus vaccines used in the European Union also must meet the minimum requirements of the European Pharmacopoeia to become licensed [13]. There have been a few attempts to compare the efficacy of different oral rabies virus vaccines in the field as executed for France and Estonia [58, 59]. However, to explain differences in the performance of ORV programs simply by the use of different vaccines is questionable, in particular if important background information, e.g. overall epidemiological situation, initial rabies incidence, topographical features, previously applied control measures, and differences in strategic ORV parameters, for both respective and adjacent areas was not considered appropriately.

The complexity of these parameters is illustrated by the use of V-RG, a recombinant vaccine manufactured by a French and a Ukrainian company. Whereas V-RG proved to be highly efficacious in Western Europe [60], in the Ukraine there has been no effect on rabies prevalence over the past 15 years despite the distribution of 50 million vaccine doses over that time period [www.who-rabies-bulletin.org]. The reason for these differences is not known. Since the two different commercial products are based on the same principle, next to the factors mentioned above, differences in production-related or product-specific parameters cannot be excluded. Our data suggest a high spatial and temporal overlap of the various oral rabies virus vaccines distributed across Europe (Fig 5D, Tables 2 and 3). Even in areas exclusively vaccinated with only a single vaccine virus strain (Table 1) ORV programs were implemented at different time points (Fig 1). This complicates a comparative analysis of vaccine efficiency in the field.

### Conclusions

We show that the extent to which oral rabies virus vaccines were used in Europe varies considerably in space and time. Next to the type of vaccine virus strains used, there is significant difference in the number of vaccine doses distributed in ORV campaigns as well as tremendous spatial and temporal overlap. Although SAD derived vaccine virus strains are the most widely used, the success of ORV campaigns in Europe cannot be attributed to a single oral rabies virus vaccine or a specific group of vaccines (e.g. first vs. second generation vaccines or modified live vs. recombinant vaccines). Result are likely due to the interaction of different key components, programs and strategies, including adequate distribution of efficacious vaccine-baits that led to the elimination of fox rabies from vast areas of Europe [13].
